# Host Plant Induced Variation in Gut Bacteria of *Helicoverpa armigera*

**DOI:** 10.1371/journal.pone.0030768

**Published:** 2012-01-26

**Authors:** Natarajan Gayatri Priya, Abhishek Ojha, Mayur K. Kajla, Anand Raj, Raman Rajagopal

**Affiliations:** 1 Department of Zoology, University of Delhi, Delhi, India; 2 International Centre for Genetic Engineering and Biotechnology (ICGEB), New Delhi, India; 3 National Dope Testing Laboratory, New Delhi, India; 4 Department of Entomology, University of Wisconsin, Madison, Wisconsin, United States of America; University of Hyderabad, India

## Abstract

*Helicoverpa* are important polyphagous agricultural insect pests and they have a worldwide distribution. In this study, we report the bacterial community structure in the midgut of fifth instar larvae of *Helicoverpa armigera*, a species prevalent in the India, China, South Asia, South East Asia, Southern & Eastern Africa and Australia. Using culturable techniques, we isolated and identified members of *Bacillus firmus*, *Bacillus niabense*, *Paenibacillus jamilae*, *Cellulomonas variformis*, *Acinetobacter schindleri*, *Micrococcus yunnanesis*, *Enterobacter* sp., and *Enterococcus cassiliflavus* in insect samples collected from host plants grown in different parts of India. Besides these the presence of *Sphingomonas*, *Ralstonia*, *Delftia*, *Paracoccus* and *Bacteriodetes* was determined by culture independent molecular analysis. We found that *Enterobacter* and *Enterococcus* were universally present in all our *Helicoverpa* samples collected from different crops and in different parts of India. The bacterial diversity varied greatly among insects that were from different host plants than those from the same host plant of different locations. This result suggested that the type of host plant greatly influences the midgut bacterial diversity of *H. armigera*, more than the location of the host plant. On further analyzing the leaf from which the larva was collected, it was found that the *H. armigera* midgut bacterial community was similar to that of the leaf phyllosphere. This finding indicates that the bacterial flora of the larval midgut is influenced by the leaf surface bacterial community of the crop on which it feeds. Additionally, we found that laboratory made media or the artificial diet is a poor bacterial source for these insects compared to a natural diet of crop plant.

## Introduction

The gut represents a dynamic food assimilation system and has been the primary improvement during evolution at the branching between plants and animals. While plants evolved to manufacture their food by photosynthesis, animals took the route of extracting nutrients by digesting the consumed food produced by plants. Food consumed by animals, principally polymers of sugars, fatty acids, amino acids etc need to be metabolized to their respective easily absorbable fundamental units. Recent studies from analysis of animal genomes have concluded that animals do not possess the entire metabolic repertoire to efficiently extract the maximum of nutrients from their food and they depend on their gut microbial community for this purpose [1].

Gut bacteria are unique in the sense that they can thrive in the hostile environment of gut, withstanding extremes of pH and ionic composition and steep redox gradations. They multiply at a faster rate within the gut, than when placed *in-vitro*. Moreover, different microbes have distinct niches within the gut, the reasons and roles of which are not yet clear [2]. Study of the diversity and identity of these complex microbial communities native to the gut has been made possible by recent advances in molecular biology techniques, including sequencing of genomes. These findings help us to appreciate the concept of ‘Microbiome’, wherein, microbes also constitute a part of the host's functional genomic repertoire due to their influence on the host's physiology.

Recently, a complex microbial diversity in the gut has been reported [3], which changes comparatively under different physiological and pathological states of the host [4], [5]. Among insects, termite gut bacteria have been studied in greater detail and recently bacteria from intestinal tracts of coleopteran [6], collembolans, dipterans [7], have also been reported. Lepidopteran insects are one of the most diversified insect groups [8] that are exclusively phytophagous and consequently expected to have an efficient gut microbial community to enable digestion of the cellulosic food material. Among the different species of polyphagous *Helicoverpa*, *Helicoverpa armigera* inhabits diverse ecological habitats and is the most important insect pest occurring in the developing world that causes heavy yield losses of a diverse range of dicot and monocot crops [9]. In mosquito and gypsy moth, variation in gut microbial fauna appears to depend on the ecological niche and the geographical location of the host [10], [11]. A previous study on *H. armigera* showed differences in bacterial communities of field caught and lab reared populations [12]. However, the diversity of gut microbes in insect pest *H. armigera* has not been studied in relation to their host plants. Therefore we carried out the present study on *H. armigera* (Kingdom: Animalia, Phylum: Arthropoda, Class: Insecta, Order: Lepidoptera, Familty: Noctuidae) which is the most important agriculture crop pest. It is widely distributed in the old world attacking varied plant families including monocots like maize, sorghum, bajra and various families of dicots like cotton and bhindi (Malvaceae), sunflower (Asteraceae), groundnut, chickpea, pigeon pea (Leguminosae) potato, tomato, brinjal (Solanacae) [13]. Here, we specifically addressed the question whether the host plant type would affect the gut microbial fauna of *H. armigera* and also report the diversity of gut bacteria of the insect collected from different host plants from single location, as well as from a few host plant types from different locations in India.

## Materials and Methods

### Ethics Statement

*Helicoverpa armigera* has not been notified under any act or laws and rules thereof of the Government of India or any of the State governments of Maharashtra, Tamil Nadu, Karnataka and Delhi as an endangered or threatened species restricting or regulating its collection and observation. No permits were required, for collecting the larvae from the field since *H. armigera* is not an endangered species affecting the biodiversity status.

### Insects

The fifth instar larvae of *H. armigera* were collected in labeled plastic boxes, from agricultural fields of different plants (castor, chickpea, cotton, ladyfinger, redgram, sorghum, sunflower and tomato) growing in Pachora, India. Additionally, larvae were also collected from chickpea, cotton and tomato from Bangalore, Coimbatore and Delhi. These samples were collected in 2007 and transported to the laboratory in New Delhi, India for further studies. The overnight starved larvae were surface sterilized in 70% ethanol and their midguts dissected out under aseptic conditions. They were processed immediately for isolating culturable bacteria or stored in RNA-later solution (Qiagen) at −20°C for genomic DNA isolation.

### Culture dependent isolation of bacteria from *H. armigera* midgut

Whole midguts from individual larvae were homogenized and sonicated (at 30 Amplitude, 1 sec pulse) in 1.5 ml eppendorf tubes containing 500 µl 1× PBS (Phosphate buffered saline, pH-7.0). The midgut extract was serially diluted in tryptic soy broth (TSB) from 10^−1^ to 10^−9^ and plated on tryptic soy agar (TSA) plates and incubated for 72 h at 30°C. Bacterial colonies were monitored visually, and several unique colonies (on the basis of color, texture, shape, size and colony morphology) were picked up and re-streaked (sub-cultured thrice) on the TSA plates to obtain pure cultures for each isolate. The pure bacterial colonies were inoculated in TSB (Hi-Media Laboratories) and cultures were stored as glycerol stocks, at −80°C. Total genomic DNA from the cultured bacteria was isolated using Qiagen genomic DNA kit.

### Isolation of genomic DNA from larval midgut in culture independent method

We followed the protocol described by Broderick et al. [10] and used whole midguts from individual larvae. Briefly, guts were homogenized in 500 µl TE buffer (Tris EDTA, 10 mM, pH 8.0) and sonicated as described above, and total volume was raised to 5.37 ml with TE buffer. The suspension was mixed thoroughly with 600 µl of 10% SDS and 5 µl of 20 mg/ml Proteinase K and incubated for 1 hour at 37°C. One ml of 5 M NaCl was added to each tube, followed by CTAB (Cetyl trimethylammonium bromide) and incubated for 30 min at 65°C. The genomic DNA from a single insect was purified by extraction with phenol∶chloroform∶isoamylalcohol (25∶24∶1) and then chloroform∶isoamylalcohol (24∶1). Finally, DNA in the aqueous phase was precipitated with isopropanol and re-suspended in 100 µl of 10 mM Tris buffer (pH 8.0).

### Isolation of genomic DNA from leaf phyllosphere

In order to compare the diversity of leaf phyllosphere bacteria with that of the larval midgut, leaves along with the larvae were collected from cotton, ladyfinger, sorghum and tomato growing in Indian Agricultural Research Institute, New Delhi, India during 2011. For isolation of DNA from leaf surface bacteria we followed protocol of Suda et al [14]. To 5 g of non-shredded fresh leaf sample, 5 ml of extraction buffer (100 mM Tris-Cl pH-9, and 40 mM EDTA), 1 ml of 10% SDS and 3 ml of Benzyl chloride were added. The sample was incubated at 50°C for 15 mins with repeated mixing at regular intervals. The leaves were removed and 3 ml of sodium acetate (3 M, pH 5.2) was added to the mixture. After incubation on ice for 10 mins, the mixture was centrifuged at 6000×g, for 15 mins at 4°C. The aqueous phase was transferred to a new tube followed by addition of equal amount of iso-propanol and centrifugation at 9000×g for 15 mins at 4°C. After a brief subsequent wash with 70% ethanol, the pellet was air dried and dissolved in required volume of TE buffer (10 mM Tris-Cl and 1 mM EDTA).

### PCR, cloning and sequencing

The 16S rRNA fragment was amplified by PCR from the midgut genomic DNA using the 27F and 1492R primers. The PCR reaction was performed using 50 ng of template DNA, 7.5 pico-moles of the primers, 1 mM dNTP, 1 U Taq DNA polymerase (Qiagen) and PCR buffer. The PCR conditions were 94°C for 60 sec, 28 cycles each of 94°C for 30 seconds, 54°C for 60 sec and 72°C for 60 sec, followed by 5 min extension at 72°C. The reaction product was separated on a 0.8% agarose gel and eluted from the gel using Qiagen gel extraction kit, ligated into pGEM-T Easy vector (Promega) and transformed into DH5α strain of *Escherichia coli*. The transformed colonies (90 clones) were checked for the presence of insert by colony PCR. The plasmid DNA was isolated from the insert positive colonies using plasmid DNA isolation kit (Real Biotech Corp) and commercially sequenced by using the T7 and SP6 vector primers, at Macrogen Inc. South Korea. All chemicals were from Sigma Aldrich and Co. until otherwise specified.

### T-RFLP analysis of the gut bacterial diversity

Restriction Enzyme Piker (REP-k) was used to select polymorphic enzymes capable of distinguishing all the bacteria identified in the 16S rRNA clone library by analyzing the full length sequences. The 16SrRNA fragment was amplified by PCR (following the same protocol as described earlier for studying bacterial diversity using culture independent method, but using a fluorescent labeled 27F primer) from individual larvae collected from different locations and different crops. This was followed by digestion with restriction enzyme, *BfaI* (MBI Fermentas). The digestion mixture contained 7 µl PCR product, 1 µl enzyme, 3 µl buffer and the total volume was made up to 30 µl with distilled water. Desalting of 5 µl mixture was done by making the volume up to 20 µl with distilled water followed by addition of 50 µl of 100% chilled ethanol and 2 µl of Sodium acetate (3 M, pH 5.2). It was pelleted down at 10,000 rpm for 20 min. The pellet was washed with 70% ethanol under the same conditions. The desalted pellet was dried and dissolved in 5 ul distilled water. About 0.5 µl of the desalted, digested mixture was mixed with 9.25 µl of Hi-di formamide and 0.25 µl of internal size standard (Genescan- 500LIZ), denatured for 5 min at 95°C and kept on ice till it was loaded into an auto sampler. The samples were analyzed by capillary electrophoresis in an ABI PRISM DNA sequencer (model 3100, Avant Gene analyzer) and the data from individual samples were analyzed with Gene Mapper software v2.0. T-RFLP profiles of two replicate samples were aligned and a pair wise comparison analysis was done in order to determine the similarity of microbial composition in the intestinal tract.

### Statistical analysis

All 16s rRNA sequences were compiled using Mac Vector (version 7.0) software suite (Oxford Molecular Group, Oxford, UK) and compared to available database entries using BLAST analysis [15]. The sequences were tested for possible chimeric structures using RDP chimera check program at Ribosomal Database Project (rdp8.core.msu.edu/cgb/chimera.cgi/su = SSU). EzTaxon server version 2.1 was used to find the sequence similarity with nearest type strains for phylogenetic tree construction. Treecon software was used to construct phylogenetic trees.

For pairwise analysis, similarity values, S*ab*, were determined by using equation 2N*ab*/(N*a*+N*b*), where N*ab* is the number of peaks in common between the samples and N*a* and N*b* are the number of total peaks in each sample. Correspondence analysis, to compare the T-RFs, was performed using MVSP 3.13r software.

## Results

### Isolation and characterization of bacteria from *H. armigera* larval midgut by culturable method

**Figure 1:**
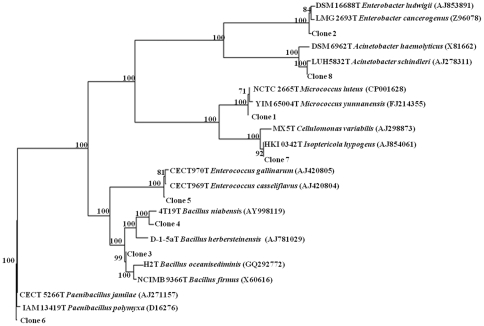
Rooted phylogenetic tree constructed for partial 16S rRNA gene of isolates cultured from *Helicoverpa armigera* gut samples. A neighbor-joining analysis with Jukes–Cantor correction and bootstrap support was performed on the gene sequences. Bootstrap values are given at nodes. Entries against (j) represent generic names and accession numbers (in parentheses) are from public databases. Entries from this work are represented as: clone number and accession number (in parentheses).

To study the bacteria associated with *H. armigera*, we first chose larvae growing on cotton as it is a major commercial crop of the country. The larval midgut contents were serial diluted in Tryptic Soy Broth (TSB) and plated on Tryptic Soy Agar (TSA) plates. Twenty seven unique colonies were isolated adopting routine microbiological techniques. These unique isolates were further screened using morphological and biochemical procedures like Gram's stain, motility, oxidase test, starch hydrolysis, nitrate reduction, sensitivity to several antibiotics *viz*. ampicillin, bacitracin, carbenicillin, cefatoxime, chloramphenicol, cephalothin, clindamycin, doxycycline, erythromycin, gentamycin, kanamycin, nalidixic acid, novobiocin, oxacillin, penicillin G, rifampicin, streptomycin, tetracycline, trimethoprim and vancomycin. Finally, based on the results of these tests (Table S1) the original 27 colonies isolated from the gut of *H. armigera* 5^th^ instar larvae were narrowed down to 8 unique bacterial colonies which were further identified. 16s rRNA sequencing and BLAST analysis (Easy Taxon) further confirmed the identity of these colonies (Table 1). The most abundant bacterial group was the low G+C Gram +ve *Firmicutes* (and within them, the genus *Bacillus* and *Enterococcus* was the maximum) followed by the Proteobacterial members *Enterobacter* and *Acinetobacter*. Actinobacterial member *Cellulomonas* was represented the least among the bacteria isolated (Table 1). The phylogenetic relatedness among these bacteria (based on their 16s rRNA sequences) is depicted in Figure 1.

**Table 1:** Phylogenetic affiliation of bacteria isolated by culturing method from 5^th^ instar larval midguts of *H. armigera* reared on cotton leaves based on complete 16S rRNA sequence.rRNA sequence.

S.No	Genus	Number of clones identified	Bacterial division	Nearest Match	Accession No.	% Similarity	Population count
**1**	*Micrococcus*	3	γ-Proteobacteria	*M. yunnanensis*	FJ214355	99.789	0.3×10^2^
**2**	*Enterobacter*	4	γ-Proteobacteria	*E. cancerogenus*	Z96078	99.252	5.3×10^7^
**3**	*Bacillus*	6	Firmicutes	*B. firmus*	X60616	99.753	3.6×10^4^
**4**	*Bacillus*	3	Firmicutes	*B. niabense*	AY998119	98.850	3.6×10^4^
**5**	*Enterococcus*	4	Firmicutes	*E. cassiliflavus*	AJ420804	99.724	2.4×10^8^
**6**	*Paenibacillus*	3	Firmicutes	*P. jamilae*	AJ271157	99.866	6.3×10^3^
**7**	*Cellulomonas*	1	Firmicutes	*C. variformis*	AJ298873	100	2×10^2^
**8**	*Acinetobacter*	3	γ-Proteobacteria	*A. schindleri.*	AJ 278311	99.657	5.8×10^5^

### Identification of bacteria from *H. armigera* larval midgut by non-culturable method

**Figure 2:**
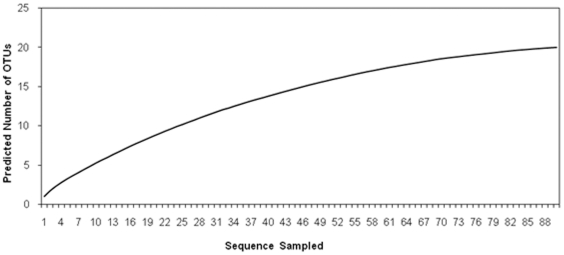
Rarefaction analysis: Clone library from the midgut of *H. armigera* was analyzed by the software DOTUR for constructing the rarefaction curve. The predicted numbers of OTU's were calculated at the 5% level of sequence divergence, to yield the curve which signifies the extent of coverage of the different bacterial genera in *H. armigera* midgut.

**Figure 3:**
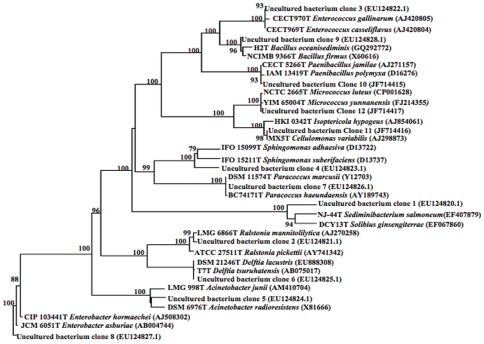
Rooted phylogenetic tree constructed for partial 16S rRNA gene of non-culturable bacteria in *Helicoverpa armigera* gut samples. A neighbor-joining analysis with Jukes–Cantor correction and bootstrap support was performed on the gene sequences. Bootstrap values are given at nodes. Entries against (j) represent generic names and accession numbers (in parentheses) are from public databases. Entries from this work are represented as: clone number and accession number (in parentheses).

Culture independent molecular analysis of bacterial diversity was performed from 16S rRNA gene library in DH5α *E. coli* strain that was obtained by cloning the 1.46 kb 16S rRNA gene amplified from *H. armigera* larval midgut in pGEM-T Easy vector. A total of 90 insert positive colonies were sequenced. Bacterial diversity analysis by DOTUR [16] predicted the presence of 29 OTUs and we were able to assign them into 12 different phylotypes resulting in coverage of 42.8%. The rarefaction curve of this clone library lacks a plateau (Figure 2), indicating that the coverage is not complete. The bacterial phylotypes recognized based on the sequence of 16S rRNA genes are listed in Table 2 and their phylogenetic relationship is depicted in Figure 3. The bacterial group encountered maximum number of times was γ-*Proteobacteria*, of which *Enterobacter* was most frequent and was represented in 53 clones (almost 59%). Apart from this, α and β *Proteobacteria* were also detected and both groups were represented by 3 clones each. The next major group was the *Firmicutes*, among which *Enterococcus* was most dominant. Actinobacterial groups, represented by *Cellulomonas* and *Micrococcus* were detected once each within the library.

**Table 2:** Phylogenetic affliation of bacteria identified by culture independent analysis from 5^th^ instar larvae of midguts of *H. armigera* reared on cotton leaves based on complete 16S r RNA.

Clone	Accession number	Representation in the library	Nearest match	Bacterial division	Accession number
**1**	EU124820	2	*Bacteriodetes*	Firmicutes	EF067680
**2**	EU124821	1	*Ralstonia mannitolillytica*	β-Proteobacteria	AJ270258
**3**	EU124822	15	*Enterococcus casseliflavus*	Firmicutes	AJ420804
**4**	EU124823	1	*Sphingomonas sp.*	α-Proteobacteria	D13737
**5**	EU124824	7	*Acinetobacter radioresistens*	γ-Proteobacteria	X81666
**6**	EU124825	2	*Delftia sp.*	β-Proteobacteria	EU888308
**7**	EU124826	2	*Paracoccus sp.*	α-Proteobacteria	Y12703
**8**	EU124827	53	*Enterobacter hormaechi*	γ-Proteobacteria	AJ508302
**9**	EU124828	2	*Bacillus sp.*	Firmicutes	X60616
**10**	JF714415	1	*Paenibacillus*	Firmicutes	AJ271157
**11**	JF714416	1	*Cellulomonas*	γ-Proteobacteria	AJ298873
**12**	JF714417	1	*Micrococcus*	γ-Proteobacteria	FJ214355

### Study of bacterial diversity among larvae from different crops and locations

After studying the bacterial diversity in the gut of *H. armigera* from cotton, we attempted to analyze the same in *H. armigera* collected from other host plants by T-RFLP analysis. The results obtained by sequencing the 16S rRNA gene library, were subjected to analysis by the program Restriction Enzyme Piker (REP-k) to select polymorphic enzymes capable of distinguishing all the bacteria identified in the library. This analysis identified one enzyme, *BfaI* as polymorphic restriction enzyme for better clarity in discriminating bacteria in gut samples. The T-RFLP profiles of the replicate samples were aligned and only peaks common to both the profiles were considered for analysis.

A comparative T-RFLP analysis of PCR-amplified 16SrRNA gene products isolated from *H. armigera*, collected from chickpea, castor, cotton, redgram, sunflower, lady finger and tomato crop fields at Pachora (Maharashtra, India) is shown in Table 3. The mean of similarity indices of the replicate larvae of all the crops was 0.910. The Dice coefficients fell in the range of 0.142 to 0.866, indicating significant variations in the bacterial communities among the samples. Pairwise comparisons showed that among the crop groups the highest coefficient (or similarity) in bacterial composition was shared by cotton and redgram (0.866). The lowest similarity in bacterial composition was between tomato and red gram (0.25). A comparison of gut bacterial diversity of larvae collected from crop plants with larvae raised on lab made artificial diet was also performed. The Dice coefficients or similarity values of artificial diet with other crops fell in the range of 0.142–0.322.

**Table 3:** Pairwise comparison for similarity of T-RFLPs from the midgut of *Helicoverpa armigera* larvae collected on different host plants from Pachora location and artificial diet.

Crops	Coefficient within group	Coefficient between crop groups							
		Chickpea	Tomato	Sorghum	Sunflower	Castor	Redgram	Ladyfinger	Cotton
**Chickpea**	0.943								
**Tomato**	0.91	0.312							
**Sorghum**	0.895	0.615	0.42						
**Sunflower**	0.932	0.755	0.41	0.50					
**Castor**	0.912	0.51	0.436	0.63	0.636				
**Redgram**	0.89	0.514	0.25	0.40	0.444	0.41			
**Ladyfinger**	0.9	0.512	0.57	0.822	0.45	0.736	0.466		
**Cotton**	0.91	0.716	0.356	0.528	0.75	0.63	0.866	0.588	
**Artificial diet**	0.9	0.322	0.2	0.142	0.312	0.2	0.272	0.214	0.214

We also conducted T-RFLP analysis in the insect samples collected from different locations *viz* Delhi (Delhi, India), Coimbatore (Tamilnadu, India), Bangalore (Karnataka, India), Pachora (Maharashtra, India) representing distinct agro-climatic regions of India. As chickpea, cotton and tomato were the crops common to all of the locations of interest, the T-RFLP profiles of *H. armigera* collected from these crops was analyzed for bacterial diversity. The mean of T-RFLP analysis between the replicate larvae of chickpea was 0.924 and the pairwise comparisons between all the locations fell in the range of 0.714–0.904 (Table 4). Similar results were obtained when analyzing cotton and tomato crops of the above mentioned locations. Larvae from cotton crops showed a similarity mean of 0.908 between the duplicate samples while the values of pairwise analysis between the locations fell in the range of 0.8–0.914 (Table 5). In larvae from tomato crops of different locations the mean was 0.911. Between different locations, the range of values for tomato crop was 0.66–0.80 (Table 6). We found that the bacterial composition was more similar between crops grown at different locations from which the larvae were collected, than between different crops.

**Table 4:** Pairwise comparison for similarity of T-RFLPs from the midgut of *Helicoverpa armigera* larvae collected from different locations on Chickpea plant.

Locations	Coefficient within group	Coefficient between Locations		
		Chickpea Pachora	Chickpea Bangalore	Chickpea Coimbatore
**Chickpea Pachora**	0.943			
**Chickpea Bangalore**	0.924	0.727		
**Chickpea Coimbatore**	0.901	0.904	0.714	
**Chickpea Delhi**	0.93	0.726	0.818	0.856

**Table 5:** Pairwise comparison for similarity of T-RFLPs from the midgut of *Helicoverpa armigera* larvae collected from different locations on Cotton plant.

Locations	Coefficient within group	Coefficient between Locations		
		Cotton Pachora	Cotton Bangalore	Cotton Coimbatore
**Cotton Pachora**	0.912			
**Cotton Bangalore**	0.90	0.80		
**Cotton Coimbatore**	0.923	0.88	0.914	
**Cotton Delhi**	0. 899	0.914	0.833	0.857

**Table 6:** Pairwise comparison for similarity of T-RFLPs from the midgut of *Helicoverpa armigera* larvae collected from different locations on Tomato plant.

Locations	Coefficient within group	Coefficient between Locations		
		Tomato Pachora	Tomato Bangalore	Tomato Coimbatore
**Tomato Pachora**	0.91			
**Tomato Bangalore**	0.932	0.666		
**Tomato Coimbatore**	0.90	0.727	0.666	
**Tomato Delhi**	0.905	0.761	0.761	0.80

Among the 12 bacterial phylotypes detected, *Enterococcus faecalis*, and *Enterobacter* sp. were the major phylotypes found in all the larvae regardless of the crop or location of samples collected including artificial diet.

**Figure 4:**
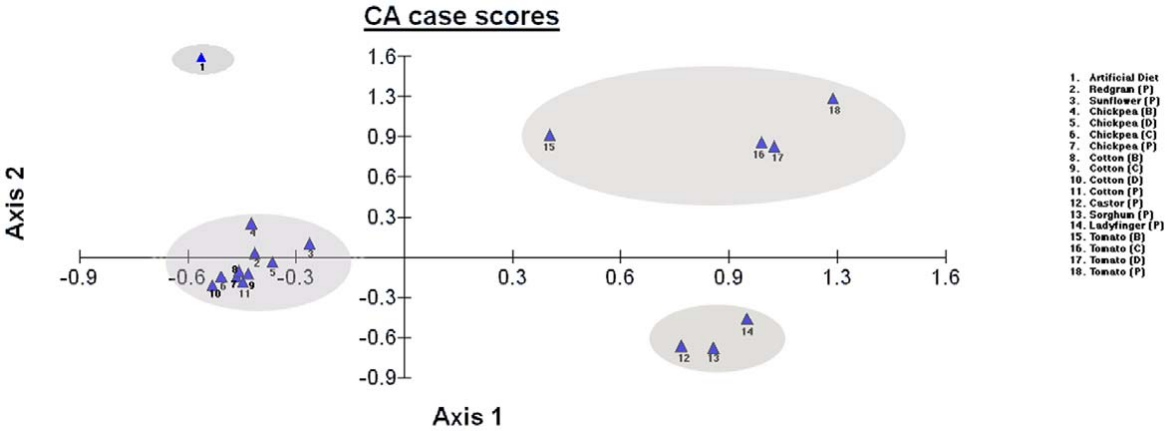
Correspondence analysis of T-RFLP data sets derived from *BfaI* digestion of 16S rDNA from gut bacterial communities of *H. armigera* of different crops collected from various locations in India. Axis 1 explains 27.93% of significance and shows the difference between tomato, ladyfinger, castor and sorghum crop plants when compared to the other crops. Axis 2 with 18.56% of significance, illustrates the difference of tomato from other crop plants and artificial diet from rest of the samples. Cluster one includes Chickpea from Pachora (P), Delhi (D), Bangalore (B) and Coimbatore (C), Cotton from P, D, B and C; Sunflower, and Redgram from Pachora. Second cluster has lady finger, sorghum and castor from Pachora. Third cluster consists of tomato crop from P, D, B and C. Artificial diet group forms the 4^th^ cluster.

We further analyzed the T-RFLP data using Correspondence analysis (CA) which is a graphical representation of similarity in a two-way contingency table. When large numbers of variables define a sample point and wish to compare all the different samples together, the CA simplifies the result by generating a statistical visualization and interpretation of the data [17]. The T-RFLP profiles of *H. armigera* larvae collected from different host plants and different locations were taken as sample points and each T-RF band defined the variables. The CA results were obtained in the form of Eigen Axis 1, which defines 27.93% variance in microbial community composition in tomato, ladyfinger, castor and sorghum crop plants when compared to the other crops, while Eigen Axis 2 defines differences between artificial diet and crop plants with 18.56% of variance. The Axis 1 and Axis 2 together explain 46.49% of variance among the sample groups. In concordance with the pairwise analysis (Tables 4, 5 and 6), we found that due to similarity in the bacterial composition among larvae collected from crops of various locations (Coimbatore, Bangalore, Delhi and Pachora) they clustered together. Pachora crops (castor, cotton, chickpea, ladyfinger, sorghum, sunflower and tomato) had significant differences amongst themselves owing to the differences in the relative abundance of individual T-RFs and were found to cluster as three separate groups (Figure 4). First cluster consisted of chickpea (all locations), cotton (all locations), redgram and sunflower. Second cluster was that of lady finger, sorghum and castor. A third separate cluster included all the locations of tomato crop. However, highest dissimilarity was found among insects raised on artificial diet with respect to insects collected from crop plants. The artificial diet group clustered very far away from other samples and thereby validating the pairwise analysis results wherein artificial diet shared least similarity values with others.

### Comparison of bacterial diversity between the leaf phyllosphere and larval midgut from different crops

The above results clearly suggest that the host plant affects bacterial diversity of *H. armigera* midgut. The question is, is this diversity because of the differences in phyllosphere bacterial diversity of different crops? Leaf phylloplane of different crops and midguts of the larvae feeding on them were analyzed by T-RFLP. Since these samples were collected from Delhi much later than the previous samples, they had to be plotted and analyzed separately. Pairwise analysis of the samples was done by calculating the values for both *H. armigera* larva and the leaf from which the larva was collected and then comparing them with each other. The Dice co-efficient values between leaf phyllosphere and larvae of different crops fell in the range of 0.73–0.93 (Table 7) indicating high levels of similarity in bacterial composition. We found that high similarity was shared between the leaf and larva samples of any crop, while a very low similarity existed amongst the crop groups.

**Table 7:** Pairwise comparison for similarity of T-RFLPs from the midgut of *Helicoverpa armigera* larvae and phyllosphere of leaves collected from different crops in Delhi.

Locations	Coefficient between samples (Leaf and larva)	Coefficient between Locations		
		Ladyfinger Delhi	Sorghum Delhi	Tomato Delhi
**Cotton Delhi**	0.833			
**Ladyfinger Delhi**	0.8	0.22		
**Sorghum Delhi**	0.93	0.727	0.181	
**Tomato Delhi**	0.73	0.33	0	0.142

**Figure 5:**
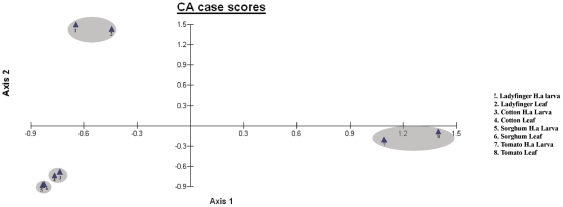
Correspondence analysis of T-RFLP data sets derived from *BfaI* digestion of 16S rDNA from bacterial communities of *H. armigera* midgut and leaf phyllosphere from different crops. Axis 1 explains 35.5% of significance and mainly showed the variation of tomato samples from other samples. Axis 2 with 32.18% of significance, illustrates the difference of ladyfinger samples from rest of the samples. Cluster one includes leaf and *H. armigera* samples, collected from the ladyfinger crop. Second cluster consists of leaf and *H. armigera* samples, collected from cotton and sorghum crops. Third cluster consists of leaf and *H. armigera* samples, collected from tomato crop.

A correspondence analysis of the data was also performed and depicted in a scatter plot. In agreement with the results of pairwise analysis, we found that different crops collected from Delhi showed a variation amongst each other and clustered separately. However, larva and leaf from the same crop showed high similarity and clustered together. This suggests that in any crop, a high degree of similarity in the bacterial composition is shared among its leaf phyllosphere and the midgut of larva feeding on it. The correspondence analysis of different crop leaves along with their respective larval data is presented in Figure 5. The Axis 1 and Axis 2 together explain 67.680% of variance among the sample groups. The CA results were obtained in the form of Eigen Axis 1 which defines 35.50% variance in microbial community composition of tomato (leaf and *H. armigera* larva) when compared to the other sample groups, while Eigen Axis 2 defines differences between ladyfinger (leaf and *H. armigera* larva) and other samples with 32.18% of the variance. Four different groups are seen, of which, ladyfinger (leaf and larva) forms the first group; cotton (leaf and larva) forms second group, sorghum (leaf and larva) clusters near cotton samples to form a third group, while tomato (leaf and larva) forms the third group (Figure 5).

## Discussion

The diversity and metabolic abilities of gut bacteria in higher animals like man is reported to be in the range of 1500–2000 different bacteria, which contribute both in number (quantitative) and diversity (quality) to the host. There are ten times more numbers of bacteria in gut when compared to the total number of cells in the host and the bacteria have more than ten times the metabolic diversity [18] when compared to the host's genome. Thus cohabilitating commensal bacteria in the gut has been a very early and essential step during evolution.

Gut microflora is shown to play a major role in controlling sexual performance, mating preferences and oviposition of the host insect [19], [20]. The influence of the gut bacteria on the insect's growth and development [18] could significantly contribute the ecological success of the host population, including their resistance to major insecticides and pesticides [21]. In order to decipher the non-pathogenic interaction between the bacteria and the host, we need to first study the gut flora of the host. Our culture dependent and culture independent studies showed that the field caught populations and the lab reared population showed significant difference in bacterial population in agreement to the previous studies [12]. This difference is due to the fact that the laboratory raised insects are exposed to narrower range of food and environmental factors compared to the field collected counterparts [11]. However a considerable difference in types of species colonizing the insect raised on artificial diet when compared to the field collected ones was observed by us. The possibility of inadequate colonizing period of the commensals can be ruled out here since we used insects that had been raised on artificial diet for 30 generation. It can be derived that the field environment exposes the insects to a wide range of microbes and provides them diet related plasticity [22].

Food availability determines the species diversity but it does not mean that the animals preferentially eating a food type are always capable to digest it by themselves. Since the survivability of animals depends on digesting the food that has been consumed, there must be other means by which the metabolism of such food products occurs. Recent studies show that several animal species rely heavily on their midgut microbial fauna for metabolizing the ingested compounds [23] toxic or not [24]. As previously described in other lepidopterans, we found *Enterobacter* and *Enterococcus* in abundance when compared to rest of the phylotypes [10], [12]. The presence of *Enterococcus* imparts the host with advantage like lowering of gut pH and providing alkaline condition which have a role in effectiveness of toxins like Bt [25]. Some of the other gut residents we identified grow under chemically diverse environmental conditions and a few of them also have the ability to degrade large molecular substances such as polycyclic aromatic hydrocarbons [26] or pesticides [27]. Furthermore, the presence of *Enterobacter* and *Enterococcus* in all the larvae regardless of the feeding substrate strongly reaffirmed their functional implications on the insect host.

It has been noted that food plant switching affects the gut bacterial composition of the host [10], [28]. We therefore asked the question whether the host plant type affects the bacterial profiles of polyphagous insects. Our results showed that the differences in bacterial composition among larvae collected from different crops in a location are highly significant (Table 3). This can be attributed to the possible disparity in nutrient content of the different crops which enable different bacteria to colonize different plant phyllosphere. The leaf surface or the phyllosphere of the host plant contains abundant bacterial flora [29] which might play an important role in shaping the commensal population of the insect feeding on the plant [30]. However, the gut bacterial composition of *Helicoverpa* on a single crop species from different agro-climatic locations, showed some similarity among themselves. It has been suggested that the difference seen in the bacterial communities between the insects collected from various locations could be due to the spatial and temporal variations in the phyllosphere community [31], [32]. The four locations analysed by us fall under different climatic zones (Bangalore has a tropical savanna type climate, Coimbatore has a moderate but pleasant climate, Delhi has a sub-tropical humid climate and Pachora has wet and dry tropical climate).

Thus it can be stated that both the host plant, as well as the plant's geographical location affects the resident gut bacterial population of the insect feeding on the crop. We then wished explore whether host plant or location has a greater role in determining gut bacterial community.

The bacterial diversity of each crop was further compared based on the presence or absence of individual T-RFs by Correspondence analysis (Figure 4). The clustering analysis brought the location groups near the same axis to form a single cluster. The clustering was found to be comparatively closer in case of the same crop from different locations whereas in case of different crops from the same location the clustering was varied. Further by the correspondence analysis, the bacterial diversity differences between crops (Table 3) could be visualized as 4 major clusters. It was noteworthy that larvae raised on artificial diet were found to cluster away from all other crop plant clusters. This was in agreement with the result from pairwise analysis of the T-RFLP profiles. Thus, the artificial diet can therefore be considered to be a poor source for the insect host with respect to the bacterial content.

As mentioned earlier, one of the factors influencing the diversity of midgut bacterial flora of the *Helicoverpa* larva could be the crop leaf surface which is also prone to microbial colonization. To investigate this, we performed a T-RFLP analysis on both the larva and the leaf phyllosphere of different crops in a location. The analysis of the T-RFs indicated that the bacterial profiles of both leaf and larva from the same crop were very similar and thereby clustered together (Table 7, Figure 5). In other words it can be stated that the larval midgut bacterial composition is a subset of the leaf phylloplane bacterial community. The differences in physiology of the crops may provide differences in bacterial communities at the leaf surface, due to which the larvae that feed on them in turn show variation amongst each other. This finding validates our previous results wherein, differences in *H. armigera* midgut bacterial community between various crops in one location, was more than the difference between various locations for a given crop. It has to be noted that the similarity in bacterial composition of larvae from cotton and sorghum crops of Delhi clusters them together. However this similarity is not seen among cotton and sorghum larvae collected from Pachora. This inconsistency could be due to the reason that the Pachora and Delhi samples were collected at two different time periods altogether.

We conclude that switching of the host plant by *H. armigera* larvae rather than the location, significantly affects its gut bacterial community. Since artificial diet is a poor source of bacteria for insects compared to natural crop plants, it can be said that the studies with insects raised on such a media need not represent the natural population present in the field especially with respect to its bacterial diversity. We also see that the leaf phyllosphere bacteria not only offers functional resistance to its host plant [33] but also influences the midgut bacterial community of the insect larva feeding on it. Further analysis of these systems may identify as to what other factors contribute to the significant variation in gut bacteria among larvae from different crops than from different locations. Investigations on the nature of adaptations that permit the resident flora to function in this extreme environment, and their role in maintaining these adaptations should also be attempted. The role of host plant in the establishment and shaping of the gut microbiota in host insects at different stages of life and the role of the resident gut microbiota in growth, development and in survival/susceptibility of the insect (by providing resistance to Bt toxin and other insecticides or pesticides during the lifetime) is an important aspect that needs to be studied in future.

## Supporting Information

Table S1 The number of bacterial isolates from *H.armigera* larvae were gram stained and subjected to basic biochemical characterization including oxidase, catalase, starch hydrolysis and nitrate reduction. In addition, antibiotic susceptibility of the bacterial strains was also performed. The result of all the tests for each colony is listed in the table.
(DOC)
