# Supplementary material for: Host Plant Induced Variation in Gut Bacteria of Helicoverpa armigera
Source: PLoS One. 2012 Jan 26;7(1):e30768. doi: 10.1371/journal.pone.0030768 (PMC3266921; doi:10.1371/journal.pone.0030768)
Supplement: Table S1 — The number of bacterial isolates from H.armigera larvae were gram stained and subjected to basic biochemical characterization including oxidase, catalase, starch hydrolysis and nitrate reduction. In addition, antibiotic susceptibility of the bacterial strains was also performed. The result of all the tests for each colony is listed in the table. (DOC) [file pone.0030768.s001.doc]

Supporting information legend:

Table S1: The number of bacterial isolates from *H.armigera* larvae were gram stained and subjected to basic biochemical characterization including oxidase, catalase, starch hydrolysis and nitrate reduction. In addition, antibiotic susceptibility of the bacterial strains was also performed. The result of all the tests for each colony is listed in the table

| **Tests** | **Levels** | M. luteus **(S1-2)** | **Enterobacter sp. (S1-3)** | **Enterococcus (S2-2)** | **B. niabense**  **(S2-3)** | Paenibacillus Sp. (S2-5) | Cellulomonas  Sp. (S2-6) | **Acinetobacter**  **Sp. (CL1)** |
| --- | --- | --- | --- | --- | --- | --- | --- | --- |
| Colony Morphology |  | Convex | Convex | Convex | Convex | Convex | Convex | Spherical |
| **Color** |  | Yellow | Shiny colony | Creamy | Dull white | White | Yellow | Yellow |
| **Gram Stain** |  | + | _ | + | + | + | + | _ |
| **Motility** |  | _ | + | + | + | + | + | + |
| **Shape** |  | Spherical | Rods Straight | Ovoid | Rod | Rod | Rod | Rod |
| Oxidase Test |  | + | _ | _ |  | _ | + | _ |
| Catalase Test |  | + | + | _ | + | + | + | + |
| **Starch hydrolysis** |  | _ | _ | _ | + | + | + Weakly | _ |
| **Nitrate reduction** |  | _ | + | _ | _ | + | + | _ |
| **Ampicillin** | 10 mcg | R | S | S | R | S | S | S |
| **Bacitracin** | 10 Units | S | S | S | S | I | S | S |
| **Carbenicillin** | 100 mcg | S | S | S | I | S | S | S |
| **Cefotaxime** | 30 mcg | S | S | S | I | S | S | S |
| **Chloramphenicol** | 30 mcg | I | S | I | S | S | S | S |
| **Cephalothin** | 30 mcg | S | S | S | I | S | Not tested | Not Tested |
| **Clindamycin** | 2 mcg | S | I | R | I | S | Not tested | Not Tested |
| **Doxycycline Hydrochloride** | 30 mcg | S | S | S | S | S | S | S |
| **Erythromycin** | 15 mcg | R | I | R | S | S | S | S |
| **Gentamycin** | 10 mcg | S | S | R | S | S | S | S |
| **Kanamycin** | 30 mcg | S | S | R | S | S | R | I |
| **Nalidixic acid** | 30 mcg | R | S | R | S | S | R | R |
| **Novobiocin** | 30 mcg | S | S | S | S | S | R | S |
| **Oxacillin** | 1 mcg | S | S | S | S | S | Not tested | Not Tested |
| **Penicillin G** | 10 Units | I | S | I | R | I | S | I |
| **Rifampicin** | 5 mcg | S | R | R | S | I | S | S |
| **Streptomycin** | 10 mcg | S | S | R | S | S | I | R |
| **Tetracycline** | 30 mcg | S | S | S | S | S | S | S |
| **Trimethoprim** | 5 mcg | S | S | S | S | S | R | S |
| **Vancomycin** | 30 mcg | I | S | S | S | S | S | S |
